# Full genomic analysis of an influenza A (H1N2) virus identified during 2009 pandemic in Eastern India: evidence of reassortment event between co-circulating A(H1N1)pdm09 and A/Brisbane/10/2007-like H3N2 strains

**DOI:** 10.1186/1743-422X-9-233

**Published:** 2012-10-11

**Authors:** Tapasi Roy Mukherjee, Anurodh S Agrawal, Sekhar Chakrabarti, Mamta Chawla-Sarkar

**Affiliations:** 1 National Institute of Cholera and Enteric Diseases, Beliaghata, Kolkata, India

**Keywords:** Influenza A, H1N2, Full genome, Reassortment, India

## Abstract

**Background:**

During the pandemic [Influenza A(H1N1)pdm09] period in 2009-2010, an influenza A (Inf-A) virus with H1N2 subtype (designated as A/Eastern India/N-1289/2009) was detected from a 25 years old male from Mizoram (North-eastern India).

**Objective:**

To characterize full genome of the H1N2 influenza virus.

**Methods:**

For initial detection of Influenza viruses, amplification of matrix protein (M) gene of Inf-A and B viruses was carried out by real time RT-PCR. Influenza A positive viruses are then further subtyped with HA and NA gene specific primers. Sequencing and the phylogenetic analysis was performed for the H1N2 strain to understand its origin.

**Results:**

The outcome of this full genome study revealed a unique reassortment event where the N-1289 virus acquired it’s HA gene from a 2009 pandemic H1N1 virus with swine origin and the other genes from H3N2-like viruses of human origin.

**Conclusions:**

This study provides information on possibility of occurrence of reassortment events during influenza season when infectivity is high and two different subtypes of Inf-A viruses co-circulate in same geographical location.

## Background

Influenza A viruses belongs to the family Orthomyxoviridae. Orthomyxoviruses have negative-sense single-stranded RNA genomes that are segmented, allowing for reassortment and production of novel viruses. There are two major surface glycoproteins, hemagglutinin (HA) and neuraminidase (NA), that define subtypes and are important for host range, antigenicity, pathogenesis, and diagnostic detection. The H3N2 viruses were demonstrated to have acquired HA, NA, and PB1 genes of human virus origin, PA and PB2 genes of avian virus origin, and the remaining internal genes, NP, M, and NS, of swine virus origin, thus are also referred as the triple reassortant viruses (TR H3N2) [1]. The human lineage PB1, avian lineages PB2 and PA, and swine lineages NP, M, and NS found in contemporary swine viruses are referred to as the triple reassortant internal gene (TRIG) constellation [2]. Other than sporadic transmission to humans [3], classical swine influenza A viruses of the H1N1 subtype were historically distinct from avian and other mammalian influenza viruses based on host specificity, serologic type, and/or genotype [4]. H3N2 viruses were responsible for Hong Kong flu in 1968 and were reported from Maharashtra, Andhra Pradesh, Kolkata, Nepal and other regions. Comparison of the nt and aa sequences of the HA1 gene for H1N1 and H3N2 strains showed that a subset of the circulating Kolkata strains precede the WHO recommended vaccine strains by 1–2 years [5]. Prior to pandemic of 2009, neuraminidase inhibitors were rarely used for influenza prophylaxis or treatment in India which could be the probable reason for drug sensitive strains; no genetic indication of mutations conferring neuraminidase drug resistance in the H3N2 (aa119, 152, 274, 292 or/and294) or in the H1N1 (aa275) viruses was observed during 2005–08 in eastern region of India [5]. However in 2009, oseltamivir resistant seasonal H1N1 viruses emerged with H275Y substitution [6-8]. Interestingly, inspite of oseltamivir treatment during pandemic H1N1, the co-circulating influenza A(H1N1)pdm09 strains as well as the H3N2 strains did not show any mutation containing oseltamivir resistance. Again the influenza A(H1N1)pdm09 virus, with gene segments from avian, swine and human origin had spread globally within 3 months resulting in declaration of pandemic level 6 by W.H.O [9] was unique compared to previous pandemics since it had high transmission ability but low virulence compared to previous pandemic viruses of this century [10]. In Eastern India, we have observed circulation of H1N1 and H3N2 subtypes during 2005-2009 [5]. In 2010, influenza A(H1N1)pdm09 was predominant [11].

In the present study, we are reporting detection and molecular characterization of a H1N2 strain (designated as A/Eastern India/N-1289/2009); detected from a 25 years old male in North-eastern India who was suffering from fever, and acute respiratory tract infections (ARTIs) and had histories of close contact with a person suffering from influenza A(H1N1)pdm09. Since H1N2 is not common in this region, all gene segments were sequenced to characterize the virus.

## Methods

### Sample collection and screening

Nasal and/or throat swabs of hospitalized cases were referred from different hospitals in eastern and north-eastern India during the pandemic period (June 2009-Dec 2010) [12,13]. The informed consent forms and detailed case histories were recorded before collection of sample. The study was approved by the Institutional Ethical Committees, NICED (Ethical Clearance No. A-1/2009-IEC). Initial detection of influenza viruses, was carried out as described previously [14]. The H1N2 virus characterized in this study was identified from a referred sample of a patient with severe respiratory infection in Mizoram (North-eastern India).

### Full length amplification and sequencing of viral genes

For sequencing, viral genes were amplified as described earlier [14,15]. Nucleotide (nt) sequencing was carried out in an ABI Prism 3100 Genetic Analyzer (PE Applied Biosystems, Foster City, CA, USA) using gene specific primers. Nucleotide and protein sequence BLAST search was performed using the National Centre for Biotechnology Information (NCBI, National Institutes of Health, Bethesda, MD) Basic Local Alignment Search Tool (BLAST) server on GenBank database release 143.0. [16].

### Phylogenetic analysis

Pairwise sequence alignments were performed using LALIGN software. Multiple alignments were done with Clustal W program which is available at DDBJ software. Phylogenetic tree was constructed by the neighbor-joining method [17] using the MEGA program, version 4.1.

### Gene bank accession number

The nucleotide sequence data were submitted to the GenBank under the accession numbers JQ340003, JQ340004, JQ340005, JQ340006, JQ340007, JQ340008, JQ340009, and JQ340010 for HA, NA, NP, PA, PB1, PB2, Matrix and Non structural genes respectively.

## Results

During the influenza A(H1N1)pdm09 period in 2009-2010, an Inf-A positive sample (designated as A/Eastern India/N-1289/2009) was detected from a 25 years old male in North-eastern India among 2931 samples screened till date. The sample was initially classified as influenza A(H1)pdm09, though NA gene could not be amplified using N1 specific primers. To rule out mix infection in patient, primers specific for H3 and N2 were also used, H3 did not amplify, however NA gene was amplified using N2 specific primers. Full length sequencing of HA and NA gene segments confirmed the virus to be of H1N2 subtype. For further genetic analysis, the complete coding sequences of all 8 segments were determined.

**Figure 1:**
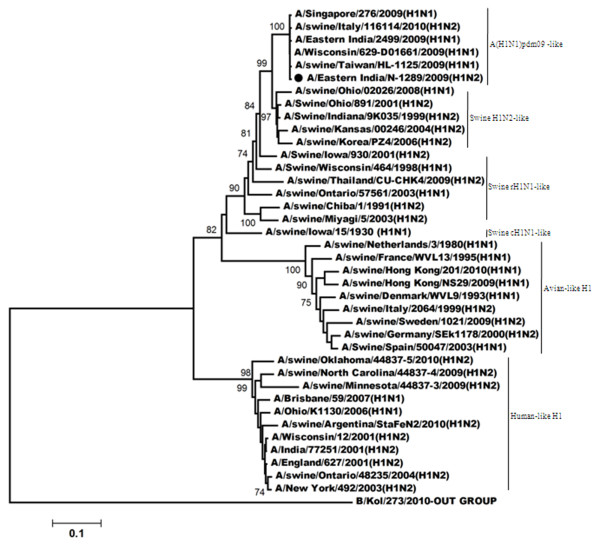
**Phylogenetic comparison based on nucleotide sequence of HA gene with that of vaccine strains and the strains from different countries.** Our strain is marked as ‘●’. The tree was rooted with cognate stretch of strain B/Kol/273/2010 for HA. The tree was created by using neighbor-joining method with 1000 bootstrap replicates.

Figure 1 showed phylogenetic relationship of HA (Hemagglutinin) gene for the H1N2 virus. Such analysis revealed that the HA gene of this H1N2 (A/Eastern India/N-1289/2009) virus was closely related to the recent influenza A(H1N1)pdm09 viruses, however; A/India/77302/2001-the H1N2 strain isolated in India during 2001, clustered with the viruses which have human-like H1 gene. Comparison of the amino acid sequences of the HA1 region of A/Eastern India/N-1289/2009 with the vaccine strain (A/Brisbane/59/2007), the recent influenza A(H1N1)pdm09 strain (A/Eastern India/2499/2009) and a H1N2 strain (A/India/77251/2001) which was isolated previously from India [18] showed several amino acid changes; for example position 147 has an insertion of a lysine residue whereas position 150 has a threonine instead of serine of the vaccine strain, position 152 has got alanine instead of serine and both 174 and 207 has serine instead of leucine and alanine respectively (Table 1). 

**Table 1:** Comparison of amino acid (aa) sequences of the HA1 region of A/Eastern India/N-1289/2009 with that of the vaccine strain (A/Brisbane/59/2007), a A(H1N1)pdm09 strain (A/Eastern India/2499/2009), and a previously reported H1N2 strain (A/India/77251/2001)

**Influenza A strains**			**Amino acid position**		
	147	150	152	174	207
***A/Brisbane/59/2007***	-	S	S	L	A
**A/Eastern India/N-1289/2009**	K	T	A	S	S
**A/Eastern India/2499/2009**	K	T	A	S	S
**A/India/77251/2001**	-				T

In respect to the vaccine strain identical residues are indicated by dots and the ‘-’ represent deletion.

**Figure 2:**
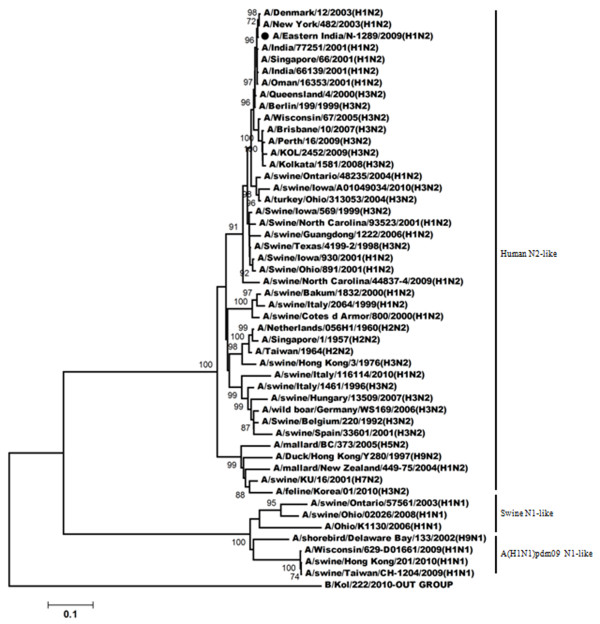
**Phylogenetic comparison based on nucleotide sequence of NA gene with that of vaccine strains and the strains from different countries.** Our strain is marked as ‘●’. The tree was rooted with cognate stretch of strain B/Kol/222/2010 for NA. The tree was created by using neighbor-joining method with 1000 bootstrap replicates.

The phylogeny of the NA gene (Figure 2) demonstrated that A/Eastern India/N-1289/2009 acquired its NA gene from triple reassortant H3N2 viruses and shares the same clade with A/Perth/16/2009 (H3N2) and with A/Brisbane/10/2007. A/India77251/2001-a H1N2 strain isolated during 2001 [18] acquired its NA gene from a H3N2 like viruses; also shares the same clade with A/Eastern India/N-1289/2009. Comparison of the amino acid sequences of NA gene of A/Eastern India/N-1289/2009 with the vaccine strain of the H3N2-like viruses (A/Brisbane/10/2007), a H1N2 strain (A/India/77251/2001) and a H3N2 strain (A/KOL/2452/2009) showed several changes at the amino acid level (Table 2). Among them two substitution were very important. One was located at the antigenic site B (E221K) and the second one was E432Q substitution. No mutation regarding resistance against neuraminidase inhibitor was observed. 

**Table 2:** A & B Comparison of amino acid (aa) sequences of the NA gene of A/Eastern India/N- 1289/2009 with that of the vaccine strain (A/Brisbane/10/2007), a H3N2 strain (A/Kol/2452/2009), and a previously reported H1N2 strain (A/India/77251/2001)

**A.**												
	**18**	**23**	**30**	**42**	**50**	**93**	**143**	**150**	**194**	**216**	**221**	**267**
***A/Brisbane/10/2007***	S	F	I	F	V	D	V	R	I	V	E	T
**A/Eastern India/N-1289/2009**	A	L	V	C	E	N	G	H	V	G	K	L
**A/India/77251/2001**	A	L	V	C	E	N	G	H	V	G	K	L
**A/KOL/2452/2009**	-	-	-									
**B.**												
					**307**	**310**	**312**	**370**	**372**	**385**	**387**	**432**
***A/Brisbane/10/2007***					I	H	T	S	L	N	K	E
**A/Eastern India/N-1289/2009**					V	Y	I	L	S	K	N	Q
**A/India/77251/2001**					V	Y	I	L	S	K	N	Q
**A/KOL/2452/2009**							I					

In respect to the vaccine strain identical residues are indicated by dots and The ‘ - ’ represent deletion.

**Figure 3:**
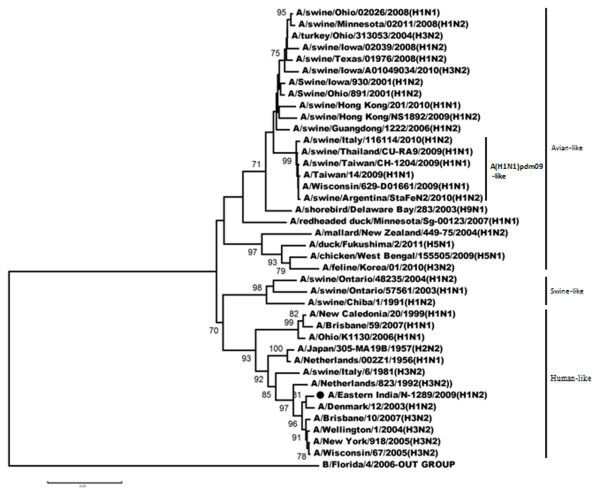
**Phylogenetic comparison based on nucleotide sequence of PB2 gene with that of vaccine strains and the strains from different countries.** Our strain is marked as ‘●’. The tree was rooted with cognate stretch of strain B/Florida/4/2006 for PB2. The tree was created by using neighbor-joining method with 1000 bootstrap replicates.

**Figure 4:**
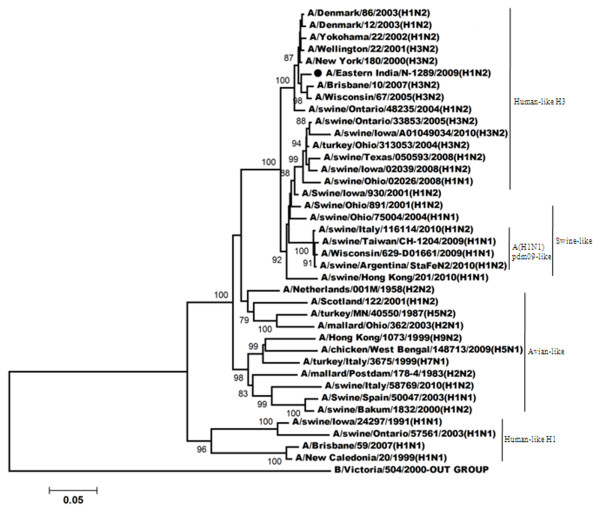
**Phylogenetic comparison based on nucleotide sequence of PB1 gene with that of vaccine strains and the strains from different countries.** Our strain is marked as ‘●’. The tree was rooted with cognate stretch of strain B/Victoria/504/2000 for PB1. The tree was created by using neighbor-joining method with 1000 bootstrap replicates.

**Figure 5:**
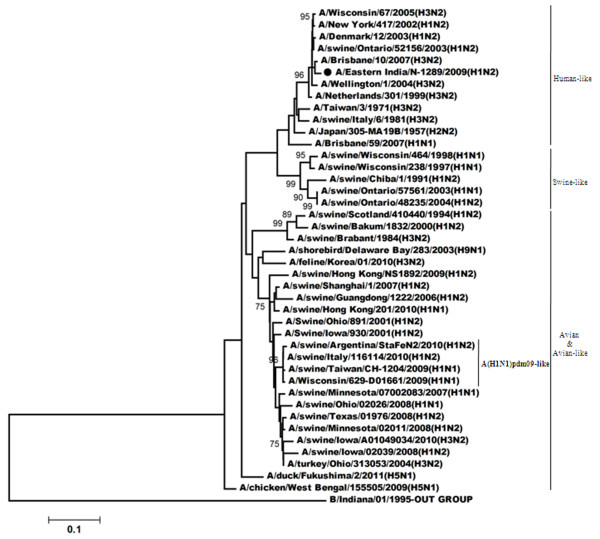
**Phylogenetic comparison based on nucleotide sequence of PA gene with that of vaccine strains and the strains from different countries.** Our strain is marked as ‘●’. The tree was rooted with cognate stretch of strain B/Indiana/01/1995 for PA. The tree was created by using neighbor-joining method with 1000 bootstrap replicates.

**Figure 6:**
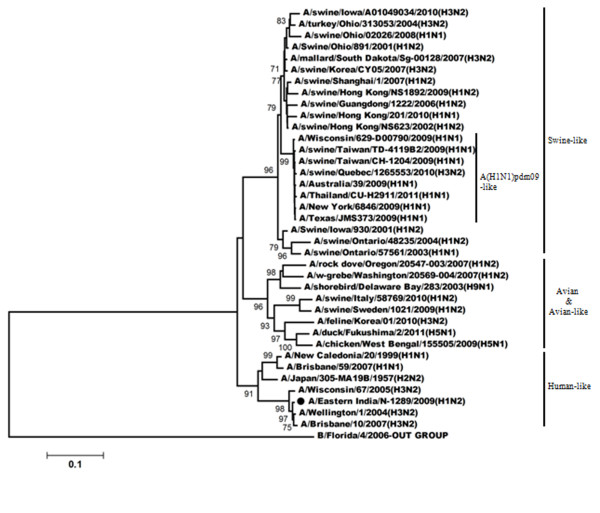
**Phylogenetic comparison based on nucleotide sequence of NP gene with that of vaccine strains and the strains from different countries.** Our strain is marked as ‘●’. The tree was rooted with cognate stretch of strain B/Florida/4/2006 for NP. The tree was created by using neighbor-joining method with 1000 bootstrap replicates.

Figures 3, 4, 5, 6 shows the phylogenetic relationship of the basic polymerase 2 gene (PB2), basic polymerase 1 gene (PB1), acidic polymerase gene (PA) and nucleoprotein gene (NP) respectively. Our result demonstrated that PB2, PB1, PA and NP genes of A/Eastern India/N-1289/2009 are closely related to A/Brisbane/10/2007 (the vaccine strain of H3N2-like viruses) but distantly related to A/Brisbane/59/2007 (the vaccine strain of H1N1-like viruses). Comparison of the amino acid sequences with A/Brisbane/10/2007 revealed that there were altogether 5 positions in PB2 gene, 11 positions in PB1 gene and 3 positions in NP gene where amino acid changes were observed; moreover no change was observed in the amino acid sequences of the PA gene (Additional file 1: Table S1).

**Figure 7:**
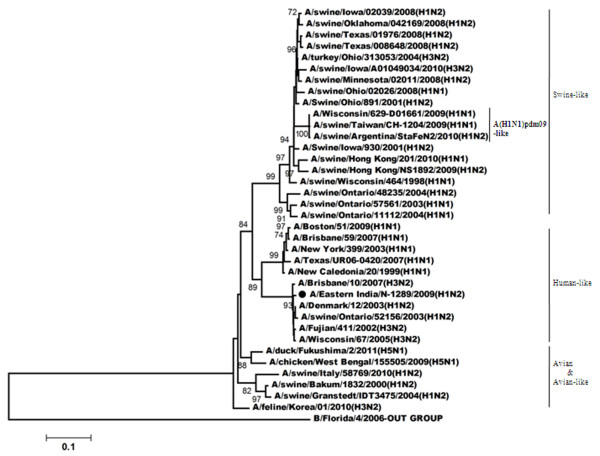
**Phylogenetic comparison based on nucleotide sequence of non**-**structural gene with that of vaccine strains and the strains from different countries.** Our strain is marked as ‘●’. The tree was rooted with cognate stretch of strain B/Florida/4/2006 for HA. The tree was created by using neighbor-joining method with 1000 bootstrap replicates.

**Figure 8:**
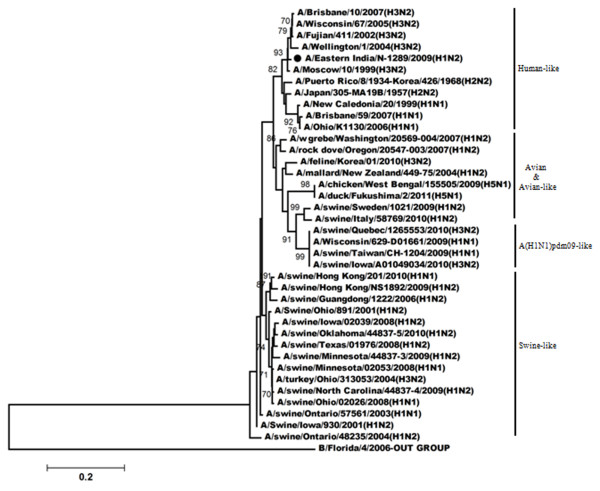
**Phylogenetic comparison based on nucleotide sequence of matrix protein gene with that of vaccine strains and the strains from different countries.** Our strain is marked as ‘●’. The tree was rooted with cognate stretch of strain B/Florida/4/2006 for HA. The tree was created by using neighbor-joining method with 1000 bootstrap replicates.

The non-structural gene also shares the same clade with the current circulating H3N2 strains and also with the vaccine strain A/Brisbane/10/2007 (Figure 7). Similarly the matrix protein gene of A/Eastern India/N-1289/2009 strain is highly homologous to H3N2 strains like A/Brisbane/10/2007 (the vaccine strain), A/Wisconsin/67/2005, and A/Fujian/411/2002 etc (Figure 8). A single amino acid change was observed in matrix protein gene as well as in non-structural gene in comparison to A/Brisbane/10/2007 (Additional file 1: Table S1). No mutation regarding amantadine resistance in the matrix protein gene was observed.

## Discussion

Influenza A viruses of the H1N2 subtype were isolated previously from humans in India [18] and Japan [19] during the 2001–2002 influenza season. Genetic analysis revealed that they were reassortants of the human H1N1 and H3N2 viruses and that they were apparently distinct from the H1N2 swine influenza viruses. However, inspite of repeated attempts to isolate the recombinant virus (designated as A/Eastern India/N-1289/2009) by inoculation of susceptible cells as well as by egg inoculation, virus could not be isolated. The reason for failure could be loss of viability as the sample came from Mizoram (~ 1219 kms from Kolkata) by courier which took >48 hrs to deliver it in lab.

Co-circulation of both influenza A(H1N1)pdm09 and seasonal (A/H1N1, A/H3N2) influenza during 2009 and complete disappearance of seasonal A/H3N2 and A/H1N1 strains in 2010 was observed in eastern India [13]. Such co-circulation is the prime cause of the generation of genetically reassortant viruses. Our study revealed that the H1N2 virus identified during 2009 was reassortant of influenza A(H1N1)pdm09 and H3N2-like viruses of human origin. Phylogenetic analysis of the HA gene revealed that the virus has got it’s HA gene from Influenza A(H1N1)pdm09 viruses whereas the virus derived the NA gene and all other gene segments from the H3N2-like viruses (Additional file 2: Table S2). Comparison of antigenic sites of HA1 region of our strain with A/Brisbane/59/2007 (the vaccine strain; which was used to clarify whether the vaccination could provide protection against this virus), A/Eastern India/2499/2009 (one of the influenza A(H1N1)pdm09 strain) and A/India/77251/2001(an H1N2 strain isolated in 2001-02 sessions) revealed that there is an insertion of a strong basic amino acid Lysine (K) at position 147. Such insertion might affect electrostatic interactions of HA with its antibodies [20]. The substitution of one nonpolar neutral amino acid with another nonpolar neutral one will not make any difference in making the hydrophobic interaction and proper conformation into the binding pocket [21] but the S152A substitution is polar to nonpolar whereas L174S and A207S show substitution of a nonpolar aa with a polar one. It could be predicted that such shift may lead to the improper hydrophobic interaction and improper conformation into the binding pocket which may affect receptor binding. The aa sequence analysis of the NA gene show more substitutions than the HA1 region, but the important one is located at position 221 where a polar negative aa has been substituted with polar positive aa. Such positivity results in more hydrophobicity of the amino acids located in that particular region of the protein which attracts specific protein regions towards the hydrophobic region. Such type of substitution also occurred at position 18, 42, 50, 267, 370 and 372. However the effects of amino acid substitution in the internal genes are not known.

## Conclusion

The strain A/Eastern India/N-1289/2009 is a classical case of sporadic reassortment event occurring during the 2009-2010 influenza season when infectivity is high and different subtypes co-circulate.

## Abbreviations

Aa: Amino acid; Nt: Nucleotide; RT-PCR: Reverse transcriptase polymerase chain reaction.

## Competing interests

The authors declare that they have no competing interests.

## Authors’ contributions

TRM drafted the manuscript and performed phylogenetic analysis. ASA performed screening of clinical samples by RT-PCR. SC provided guidance and has given final approval of the version to be published. MCS conceived the study, provided guidance and edited manuscript. All authors read and approved the final manuscript.

## Supplementary Material

Additional file 1: Table S1A-1E Comparison of amino acid (aa) changes at different positions of the internal genes (PB2, PB1, NP, NS and Matrix protein gene) of A/Eastern India/N-1289/2009 with that of the vaccine strain (A/Brisbane/10/2007). Click here for file

Additional file 2: Table S2 Table indicating closest ancestor strain (name and host origin) and % homology using BLAST analysis. Click here for file
