# Supplementary material for: Full genomic analysis of an influenza A (H1N2) virus identified during 2009 pandemic in Eastern India: evidence of reassortment event between co-circulating A(H1N1)pdm09 and A/Brisbane/10/2007-like H3N2 strains
Source: Virol J. 2012 Oct 11;9:233. doi: 10.1186/1743-422X-9-233 (PMC3576275; doi:10.1186/1743-422X-9-233)
Supplement: Additional file 1: Table S1A-1E — Comparison of amino acid (aa) changes at different positions of the internal genes (PB2, PB1, NP, NS and Matrix protein gene) of A/Eastern India/N-1289/2009 with that of the vaccine strain (A/Brisbane/10/2007). [file 1743-422X-9-233-S1.docx]

**A.**

| Strain Name | Amino acid position of PB2 gene | | | | |
| --- | --- | --- | --- | --- | --- |
|  | 225 | 253 | 344 | 454 | 570 |
| A/Brisbane/10/2007 | A | G | K | V | A |
| A/Eastern India/N-1289/2009 | S | E | R | I | S |

**B.**

| Strain Name | Amino acid position of PB1 gene | | | | | | | | | | |
| --- | --- | --- | --- | --- | --- | --- | --- | --- | --- | --- | --- |
|  | 14 | 81 | 92 | 93 | 110 | 111 | 365 | 580 | 623 | 713 | 748 |
| A/Brisbane/10/2007 | I | N | V | L | S | C | R | L | N | I | M |
| A/Eastern India/N-1289/2009 | L | I | G | M | P | Y | K | I | D | V | T |

**C.**

| Strain Name | Amino acid position of NP gene | | |
| --- | --- | --- | --- |
|  | 63 | 142 | 323 |
| A/Brisbane/10/2007 | H | S | I |
| A/Eastern India/N-1289/2009 | Y | A | V |

**D.**

| Strain Name | Amino acid position of NS gene |
| --- | --- |
|  | 26 |
| A/Brisbane/10/2007 | E |
| A/Eastern India/N-1289/2009 | K |

**E.**

| Strain Name | Amino acid position of Matrix Protein gene |
| --- | --- |
|  | 174 |
| A/Brisbane/10/2007 | R |
| A/Eastern India/N-1289/2009 | K |

Supplementary Table 1A-1E: Comparison of amino acid (aa) changes at different positions of the internal genes (PB2, PB1, NP, NS and Matrix protein gene) of A/Eastern India/N-1289/2009 with that of the vaccine strain (A/Brisbane/10/2007).
