# Supplementary material for: Full genomic analysis of an influenza A (H1N2) virus identified during 2009 pandemic in Eastern India: evidence of reassortment event between co-circulating A(H1N1)pdm09 and A/Brisbane/10/2007-like H3N2 strains
Source: Virol J. 2012 Oct 11;9:233. doi: 10.1186/1743-422X-9-233 (PMC3576275; doi:10.1186/1743-422X-9-233)
Supplement: Additional file 2: Table S2 — Table indicating closest ancestor strain (name and host origin) and % homology using BLAST analysis. [file 1743-422X-9-233-S2.docx]

| Strain | A/Eastern India/N-1289/2009 | | | | | | | |
| --- | --- | --- | --- | --- | --- | --- | --- | --- |
|  | HA | NA | PB2 | PB1 | PA | NP | NS | Matrix |
| A/Italy/116114/10 (H1N2) | 95% | 85.1% | 82.5% | 91.7% | 83% | 79.9% | - | 84.6% |
| A/New York/482/2003 (H1N2) | 75.1% | 96.6% | 97.6% | 97.8% | 97.5% | 98.4% | 95.8% | 96% |
| A/Swine/Taiwan/HL-1125/2009(H1N1) | 92.6% | 51.3% | 83% | 92.3% | 81.4% | 81.3% | 76.3% | 82.3% |
| A/Brisbane/59/2007 (H1N1) | 73.1% | 51.2% | 60.9% | 79.7% | 83.5% | 83.8% | 85.6% | 89.2% |
| A/Brisbane/10/2007 (H3N2) | 53.4% | 95.3% | 97.2% | 97.1% | 98.5% | 97.6% | 97.5% | 98.3% |
| A/Hong Kong/HKU53/2005 (H3N2) | 54.1% | 96.6% | 96.2% | 96.2% | 97.3% | 95.3% | 98.9% | 98.8% |
| A/California/NHRC0001/2007 (H3N2) | 53.8% | 94.7% | 97.8% | 97.5% | 99% | 98.5% | 96.2% | 97% |

Supplementary Table 2: Table indicating closest ancestor strain (name and host origin) and % homology using BLAST analysis
